# Supplementary material for: Original Synthesis of a Nucleolipid for Preparation of Vesicular Spherical Nucleic Acids
Source: Nanomaterials (Basel). 2022 Oct 18;12(20):3645. doi: 10.3390/nano12203645 (PMC9609631; doi:10.3390/nano12203645)
Supplement: Supplementary file 1 [file nanomaterials-12-03645-s001.zip › nanomaterials-1951477-supplementary.pdf]

Supplementary Materials

Original Synthesis of a Nucleolipid for Preparation of Vesicular Spherical Nucleic Acids

Erik Dimitrov [1], Natalia Toncheva-Moncheva [1,*], Pavel Bakardzhiev [1], Aleksander Forys [2], Jordan Doumanov [3], Kirilka Mladenova [3], Svetla Petrova [3], Barbara Trzebicka [2] and Stanislav Rangelov [1,*]

[1] Institute of Polymers, Bulgarian Academy of Sciences, Akad. G. Bonchev St. 103A, 1113 Sofia, Bulgaria
[2] Centre of Polymer and Carbon Materials, Polish Academy of Sciences, M. Curie-Sklodowskiej 34, 41-819 Zabrze, Poland
[3] Department of Biochemistry, Faculty of Biology, Sofia University St. Kliment Ohridski, Dragan Tsankov Blvd. 8, 1164 Sofia, Bulgaria
* Correspondence: ntoncheva@polymer.bas.bg (N.T.-M.); rangelov@polymer.bas.bg (S.R.)

I. Composition and characterization of ThiolC$_6$-oligonucleotide

ThiolC$_6$-oligonucleotide is a single-stranded DNA oligonucleotide, functionalized by the thiol group. It was purchased from Biomers.net GmbH. The sequence and composition as well as characterization data, according to the producer, are presented in Table S1 and Figure S1.

Table S1. Sequence and composition of the ThiolC$_6$-oligonucleotide.

**Code**	**Oligonucleotide composition*** **Sequence (5′→3′)**	**M_w ($g.mol^{-1}$)**
ThiolC$_6$-oligo	Thiol-C$_6$ 5ta ata cga ctc act ata gg	6665

*5 – Spacer 18, phosphodiester followed by 6 ethylene glycol units, C6 = $(CH_2)_6$ groups.

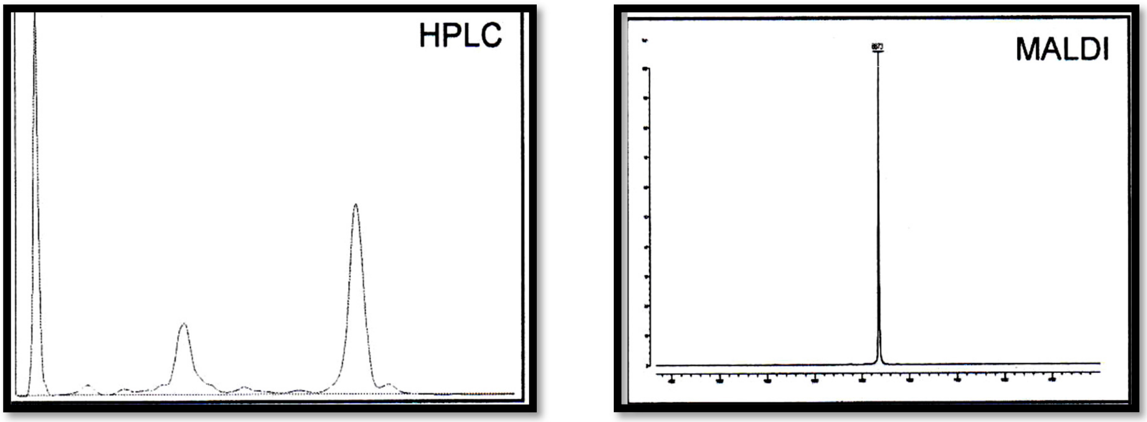

**5'-5ta ata cga ctc act ata ggg -3'**		MW Calc.	6665 g/mol
		MW Found	6673 g/mol
Length*	21	Purific.	HPLC preparative**
GC Content	38,00%	5'-Mod.:	Thiol C6
A:7,0 C:4,0 G:4,0 T/U:5,0		3'Mod	None
Ext. Coeff.:	230600	5: Spacer 18	

*ThiolC6-oligonucleotide has a typical length of 21 bases, 20 nucleotides, and 1 internal modification (Spacer18) which is marked in the sequence with the number 5.

** The preparative HPLC was used for quality control, separation, and purification of the synthesized and functionalized oligonucleotide. The isolated fraction of the main product was further analyzed by MALDI mass spectrometry. The presence of a single peak on the spectrum together with the expected molecular weight data confirmed the composition of the thiol-functionalized oligonucleotide.

Figure S1. HPLC chromatogram (left), MALDI spectrum (right), and other characterization data of ThiolC$_6$-oligonucleotide.

II. Synthesis of dihexadecyloxy-propane-2-ol (DHP)

DHP was synthesized following a procedure described elsewhere [1,2]. Details for the synthetic procedure are given below. A total of 13.068 g (0.054mol) 1-hexadecanol was placed in two-necked round bottom flask flushed with nitrogen. Then, 16.092 g (0.054 mol) of hexadecyl glycidyl ether and 17.600 g (0.0675 mol) of $SnCl_4$ were added under stirring. The stirring was maintained at 110-120˚C for 24h, then, another portion of 17.600 g of $SnCl_4$ was added and the mixture was stirred for 70 h. The quantitative conversion of hexadecyl glycidyl ether is evidenced by the lack of resonances of the epoxide protons at 2.6–3.2 ppm in the ^1H NMR spectrum of DHP (Figure S2). The product was recrystallized twice from hexane. Yield: 17.276 g, 59.24%. The synthetic pathway is presented in Figure S3.

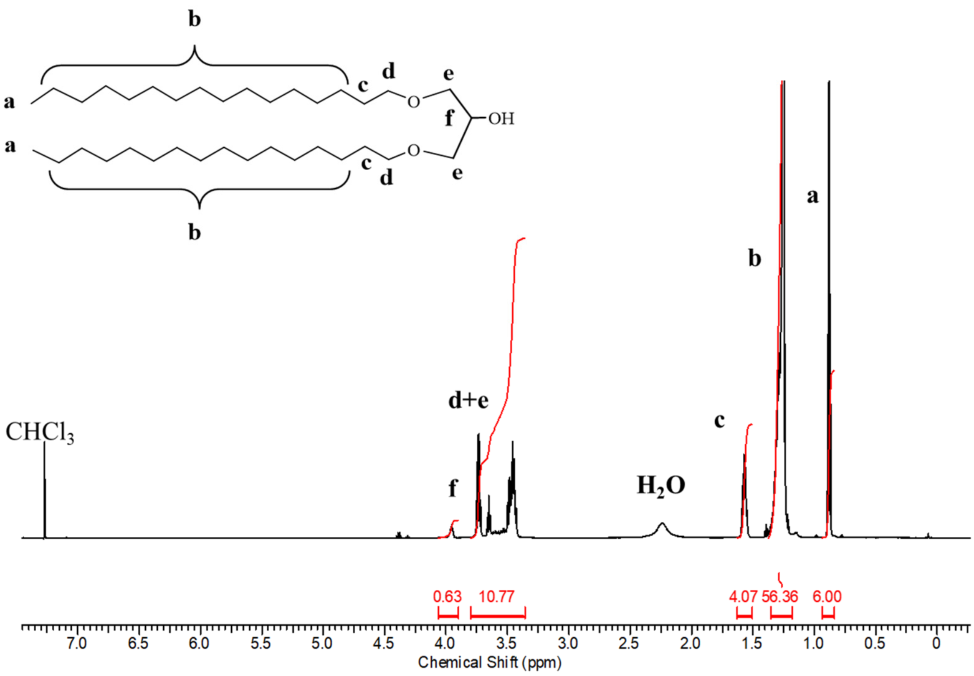

Figure S2. ^1H-NMR spectrum of 1,3-dihexadecyloxy-propane-2-ol in $CDCl_3$.

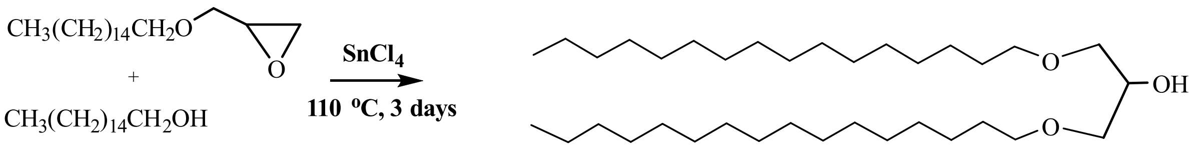

Figure S3. Schematic presentation of the synthesis of 1,3-dihexadecyloxy-propane-2-ol.

III. Synthesis of DHP-(AGE)n

A total of 1 g (1.85 mmol, 1 eq.) of DHP was freeze-dried with 3 ml of dry benzene. After removing the benzene, the flack was purged with argon, 4 ml of dry dioxane was added and the mixture was heated to 40 ˚C. The solution was treated with 0.072 g (1.85 mmol, 1 eq.) of potassium and left for 24 hours until the metal was completely dissolved. Then, 0.634 g (5.56 mmol, 3 eq.) of allyl glycidyl ether were added. The polymerization was left for 9 days at 40 ˚C after which it was terminated with 1 ml of methanol and 10 ml of dioxane were added. The resulted suspension was filtered, centrifuged, concentrated, and dried in a vacuum for 24 hours. Yield: 1.45g, 91%. Size exclusion chromatography (SEC) analysis of the purified product revealed a monomodal symmetric peak, and the calculated weight-average molar mass (M_w) and dispersity (M_w/M_n) were 1350 g.mol^{-1} and 1.1, respectively (Figure S4).

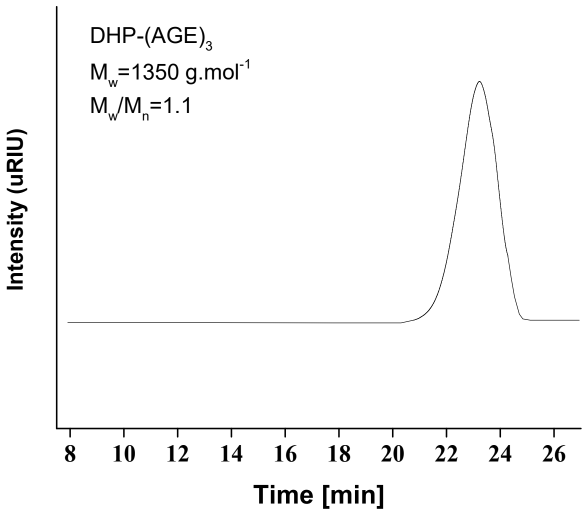

Figure S4. SEC trace of DHP-(AGE)$_3$.

IV. Description of the UV irradiation device

The custom-made irradiation device (Figure S5) is equipped with six LED-diodes as UV light sources emitting at a fixed wavelength of 365 nm. The irradiation time as well as the capacity mode—from 0.4 W (10 %) up to 4 W (100 %)—can be programmed.

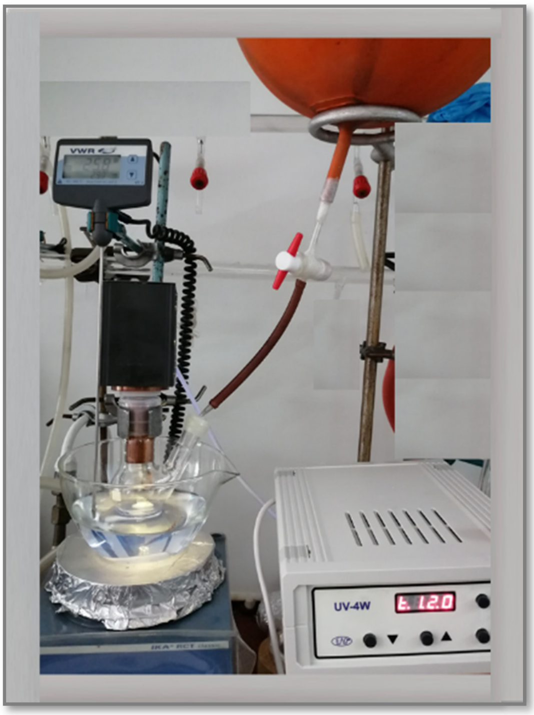

Figure S5. Photograph of the UV irradiation device.

V. Model reactions

V.1. Determination of the safe irradiation time.

A total of 100 µg of sacrificial oligonucleotides (20-25 bases long, non-specific sequence) in 2.5 mL of ultrapure water or DMF:DMSO (v/v 1:1) solvent mixture was irradiated for different time with UV light (λ = 365 nm, full capacity of 4 W of the device) in inert atmosphere at 30 °C and gentle stirring. UV-vis spectra were taken after given time intervals – 30 min, 1 h, and 3 h (Figure S6). Shifts of the peak maxima—260 nm in water and 275 nm in DMF:DMSO mixture—were not observed. The increase in the absorbance, observable in both cases for irradiation time of 3 h, indicated structural damage of the oligonucleotide strands [3,4]. On the bases of the experimental results, one may conclude that irradiation times up to 1 h are safe for the oligonucleotides and would not cause any significant structural changes. Longer irradiation times (3 h) induce damages and are harmful for the oligonucleotides.

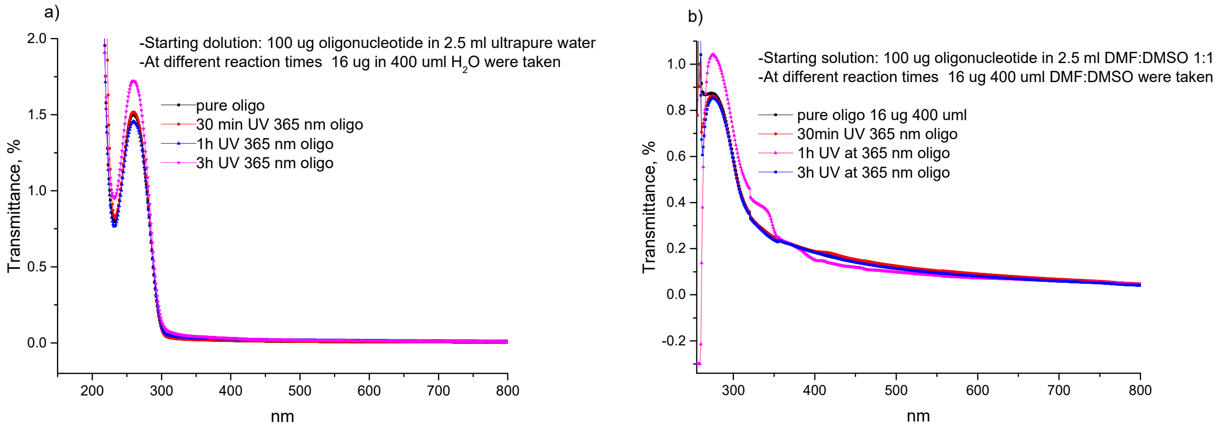

Figure S6. UV-Vis spectra of sacrificial oligonucleotides in water (a) and DMF:DMSO (v/v 1:1) solvent mixture (b) irradiated by UV light (λ = 365 nm, full capacity of 4 W of the device) in inert atmosphere at 30 °C and gentle stirring. Spectra were taken at different time intervals as indicated. Peak maxima at 260 nm and 275 nm in (a) and (b), respectively.

V.2. Conjugation of DHP-(AGE)$_n$ with 2-aminoethanethiol hydrochloride.

A total of 220 mg (0.25 mmol, 1 eq.) of DHP-(AGE)$_n$ and 85 mg (0.75 mmol, 3 eq.) of 2-aminoethanethiol hydrochloride were dissolved in 2.5 ml of a DMSO:DMF (v/v 1:1) solvent mixture in a round-bottom flask. The flask was assembled with the custom-made irradiation device (Figure S5) and the reaction mixture was irradiated for 3 h in inert atmosphere at 30 °C, full capacity of the device (4 W) and with gentle stirring. Samples for ^{1}H NMR spectroscopy were taken at specific time intervals. ^{1}H NMR spectra are shown in Figure S7. As seen from the figure, new signals at 1.75 and 2.7 ppm corresponding to methylene protons adjacent to the thioether group appeared. This was accompanied with a decrease in the intensity of the signals at 5.1, 5.2, and 5.9 ppm assigned to the allyl group. Based on the peak integral ratio of the signals for the methylene protons at 1.75 and 2.7 ppm, and those corresponding to the allyl group at 5.1, 5.2, and 5.9 ppm, we calculated that two of the three allyl groups of DHP-(AGE)$_3$ reacted with 2-aminoethanethiol hydrochloride. ^{1}H NMR data indicated that the reaction of the third allyl group did not proceed even in extended time of 3 hours. Most probably, charge repulsion between the ammonium groups prevented the reaction. The thiol-ene click reaction between DHP-(AGE)$_3$ and 2-aminoethanethiol hydrochloride is schematically presented in Figure S8.

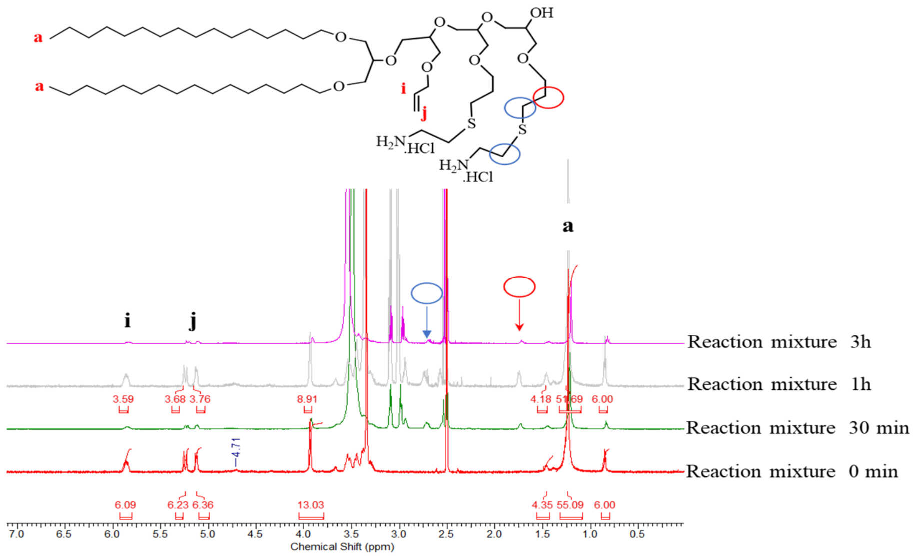

Figure S7. ^{1}H NMR spectra of the DHP-(AGE)$_3$/aminoethanethiol hydrochloride reaction mixture. Spectra were taken in DMSO-d6 at different time intervals as indicated.

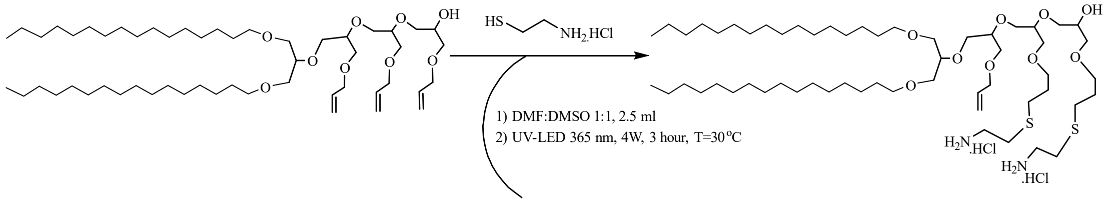

Figure S8. Schematic presentation of the coupling reaction of DHP-(AGE)$_3$ with 2-aminoethanethiol hydrochloride.

V.3. Conjugation of DHP-(AGE)$_n$ with PEGMET.

A total of 66.19 mg (0.075 mmol, 3 eq.) of DHP-(AGE)$_n$ and 50 mg of poly(ethylene glycol) methyl ether thiol (PEGMET, M_w=2000 g/mol) (0.025 mmol, 1 eq.) were dissolved in 2.5 ml of deuterated benzene in a round-bottom flask. The flask was assembled with the custom-made irradiation device (Figure S5) and the reaction mixture was irradiated for 3 h in inert atmosphere at 30 °C, full capacity of the device (4 W) and gentle stirring. Samples for ^{1}H NMR spectroscopy were taken at specific time intervals. ^{1}H NMR spectra are shown in Figure S9. As seen from the figure, the intensity of the signals for the allyl protons at 5.0–5.4 and 5.8 ppm decreased with reaction time. Based on the integral ratio of these protons with the methyl protons of the CH_3 group of PEGMET at 0.85 ppm, we calculated that one of the allyl groups reacted with the thiol group

of PEGMET for 1 hour. The thiol-ene click reaction between DHP-(AGE)3 and PEGMET is schematically presented in Figure S10.

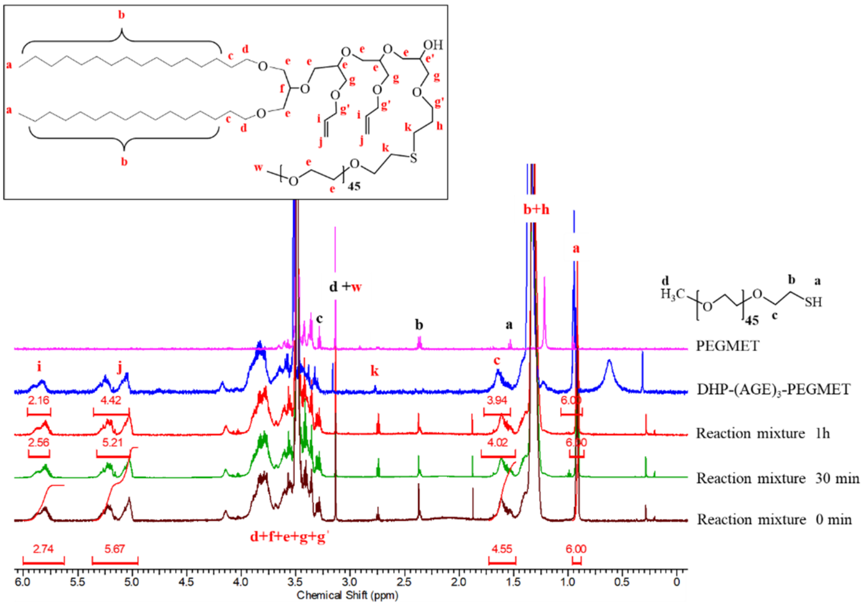

Figure S9. ^{1}H NMR spectra of DHP-(AGE)3/PEGMET reaction mixture. Spectra were taken in deuterated benzene at different time intervals as indicated.

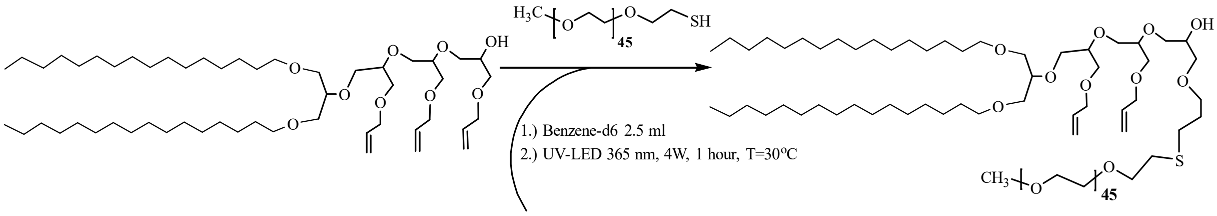

Figure S10. Schematic presentation of the coupling reaction of DHP-(AGE)3 with PEGMET.

VI. Calculation of the average number of oligonucleotide strands per vesicle and grafting density

The average number of oligonucleotide strands per vesicle was determined from the number of nucleolipid molecules and number of liposomes. Given the content and molar mass of the nucleolipid of 7509 g.mol^{-1}, and using the Avogadro's number, the total number of nucleolipid molecules was 2.41x10^{16}. The total number of liposomes (6.21x10^{12}) was determined from the total surface area of DPPC molecules, assuming an area per DPPC molecule of 0.65 nm^2 [5] (5.09x10^{17}) and the total surface area of a liposome with hydrodynamic radius of 57.1 nm (81943 nm^2). The resulting value was 3880 strands per vesicle. Finally, the area per oligonucleotide strand

in the vesicle and its reciprocal grafting density were calculated from the surface of a single vesicle and the number of strands per vesicle to be 21.12 nm^2 and 0.047 nm^{-2}, respectively. By multiplying the latter with $1x10^{14}$ and dividing by the Avogadro's number, the grafting density was converted from (nm^{-2}) into ($pmol.cm^{-2}$).

VII. *Calculation of the Flory radius, R_F, and determination of the critical grafting density (σ_{cr}) at the transition from mushroom to brush conformation*

The Flory radius, R_F, is the end-to-end distance of a free polymer coil in a theta solvent, resulting from the balance between the expanding steric forces and the counteracting entropic forces from stretching of the coils. In its simplest form it is given by equation (1) [6]:

$$R_F = an^{3/5} \qquad (1)$$

Here, *a* is the length of the monomer unit and *n* is the degree of polymerization. The hydrophilic section of the nucleolipid is composed of one hexaethylene glycol spacer and 21 bases. If the lengths of ethylene oxide unit and nucleotide unit are taken as 0.39 nm and 0.34 nm, respectively, and the total degree of polymerization is 27 (= 6 + 21), then for R_F we obtain:

$$\mathrm{R_F} = \left(\frac{6}{27} \times 0.39 + \frac{21}{27} \times 0.34\right) \times (6 + 21)^{\frac{3}{5}} = 2.53 \text{ nm}$$

The polymer coils start to interact laterally when the distance between the grafting points is equal to or less than the Flory radius. Thus, the grafting density at the transition from an unextended (mushroom) to brush conformation (σ_{cr}) can be determined by $\sigma_{cr} = 1/R_F^2$. Thus, for σ_{cr} of the nucleolipid we obtain $\sigma_{cr} = 0.156$ nm^{-2}. When $\sigma < \sigma_{cr}$ the tethered polymer coils (or oligonucleotide strands) are isolated; if $\sigma > \sigma_{cr}$ they interact laterally, overlap, and adopt a more extended (brush) conformation.

REFERENCES

1. Rangelov, S.; Petrova, E.; Berlinova, I.; Tsvetanov, Ch. Synthesis and Polymerization of Novel Oxirane Bearing an Aliphatic Double Chain Moiety. *Polymer* **2001**, *42*, 4483–4491. https://doi.org/10.1016/S0032-3861(00)00826-0.
2. Bakardzhiev, P.; Rangelov, S.; Trzebicka, B.; Momekova, D.; Lalev, G. Nanostructures by Self-Assembly of Polyglycidol-Derivatized Lipids. *RSC Adv.* **2014**, *4*, 37208–37219. https://doi.org/10.1039/C4RA03102D.
3. Basu, S.; Dasgupta, N.N. Spectrophotometric Investigation of DNA in the Ultraviolet. *Biochim. Biophys. Acta* **1967**, *145*, 391–397. https://doi.org/10.1016/0005-2787(67)90057-3.
4. Tankovskaia, S.A.; Kotb, O.M.; Dommes, O.A.; Paston, S.V. DNA Damage Induced by Gamma-Radiation Revealed from UV Absorption Spectroscopy. *J. Phys. Conf. Series* **2018**, *1038*, 012027. https://doi.org/10.1088/1742-6596/1038/1/012027.
5. Nagle, J.F.; Tristram-Nagle, S. Structure of lipid bilayers. *Biochim. Biophys. Acta* **2000**, *1469*, 159–195. https://doi.org/10.1016/s0304-4157(00)00016-2.
6. Flory, P.J. *Principles of Polymer Chemistry*; Cornell University Press: Ithaca, NY, USA, 1953.
